# MDI 301, a synthetic retinoid, depressed levels of matrix metalloproteinases and oxidative stress in diabetic dermal fibroblasts

**DOI:** 10.18632/oncotarget.16803

**Published:** 2017-04-03

**Authors:** Jianhua Zhai, Yuli Wang

**Affiliations:** ^1^ Department of Emergency, Tianjin Medical University of General Hospital, Tianjin, China; ^2^ Tianjin Institute of Pharmaceutical Research, Tianjin, China

**Keywords:** diabetic fibroblast cell, high glucose, retinoid acid, MMPs, TIMP1

## Abstract

Diabetic foot ulcerations could result in serious consequences such as amputations. The up-regulation of matrix metalloproteinases and down-regulation of TIMP1 were remarked as distinctive biological characteristics in the diabetic dermal fibroblast. The current study was performed in order to clarify the effect of high glucose on formation of diabetic dermal fibroblast cell. In addition, the effect of MDI 301 on ameliorating diabetic fibroblasts was investigated in this study. The mRNA and protein expression levels of MMPs, TIMP1 and catalase were evaluated against fibroblasts treated with high glucose (30 mM) using qRT-PCR, western blotting and zymography assays. Methods were also employed for investigating the biological effects of MDI 301 on high glucose-induced diabetic fibroblasts. In this study, we found that the unbalance of oxidative stress induced by high glucose concentration play an important role in the formation of diabetic dermal fibroblast from normal cells. In addition, MDI 301, a picolinic acid-substituted ester of 9-cis retinoic acid was employed in this study in order to ameliorate symptoms on diabetic dermal fibroblast induced by high glucose concentration. We found MDI 301 alleviate the effects of high glucose-induced skin damage by balancing the oxidative stress and regulating the MMPs and TIMP1 levels. Our finding indicated that MDI 301 offers the potential for repairing the faulty skin function arising from diabetes.

## INTRODUCTION

Diabetic foot ulceration is a contributing factor in some of the 38,500 amputations performed each year [1]. Diabetic foot ulceration, as one of the more common and serious complications of diabetes, remains a cause of considerable morbidity despite the early identification of foot insensitivity, vascular insufficiency, and deformities [2]. The reduced procollagen synthesis and increased levels of connective tissue-degrading matrix metalloproteinases (MMPs) have been identified as important factors in the early diagnosis of diabetic foot ulcerations [3–6]. TIMP1, tissue inhibitor of metalloproteinase-1, plays an important role in balancing the TIMP1/MMPs ratios [3, 7] which may represent potential biomarkers for predicting the clinical morbidity of diabetic foot ulceration patients as well [5]. In addition, Elida González demonstrated a model explaining the relationship between MMP activities and oxidative stress in diabetic rats [8]. Her data demonstrated the links between oxidative stress and alterations in the developmental pathways in which MMPs are involved in diabetic dermal fibroblast [8]. According to previous finding, capable of inducing oxidative stress and mitochondrial dysfunctions in neurons, high glucose levels may also elicit a similar effect in diabetic fibroblasts [9].

MDI 301 is a picolinic acid-substituted ester of 9-*cis* retinoic acid that shows biological activity on skin damaged by ultraviolet radiation [10, 11]. MDI 301 increases epidermal thickness, procollagen synthesis and decreases matrix metalloproteinase (MMP) activity in organ-cultured skin [10]. Unlike all-*trans* retinoic acid (RA), MDI 301 does not induce the expression of proinflammatory cytokines or induce the expression of leukocyte adhesion molecules in human skin [10, 11]. It has also been reported that treatment of aged skin with MDI 301 stimulates the proliferation of dermal fibroblasts and reduces the expression of a wide range of MMPs including MMP-1, MMP-3 and MMP-9 [10]. According to its biological activities, MDI 301 was identified as a potential clinical drug in the treatment of diabetic foot ulcerations.

In the present study, the biological effects of MDI 301 on diabetic fibroblasts induced by high glucose concentration were investigated since high glucose-induced skin damage is more possible reason than ultraviolet radiation-induced skin damage for diabetic patients. Our findings demonstrated initially that high glucose levels can induce an oxidative stress-mediated morbidity of healthy fibroblasts. In addition, MDI 301 is capable of alleviating oxidative stress in diabetic fibroblasts. Results indicated that MDI 301 might be potent to ameliorate the diabetic skin damage.

## RESULTS

### The effects of high glucose on normal fibroblasts

**Figure 1:**
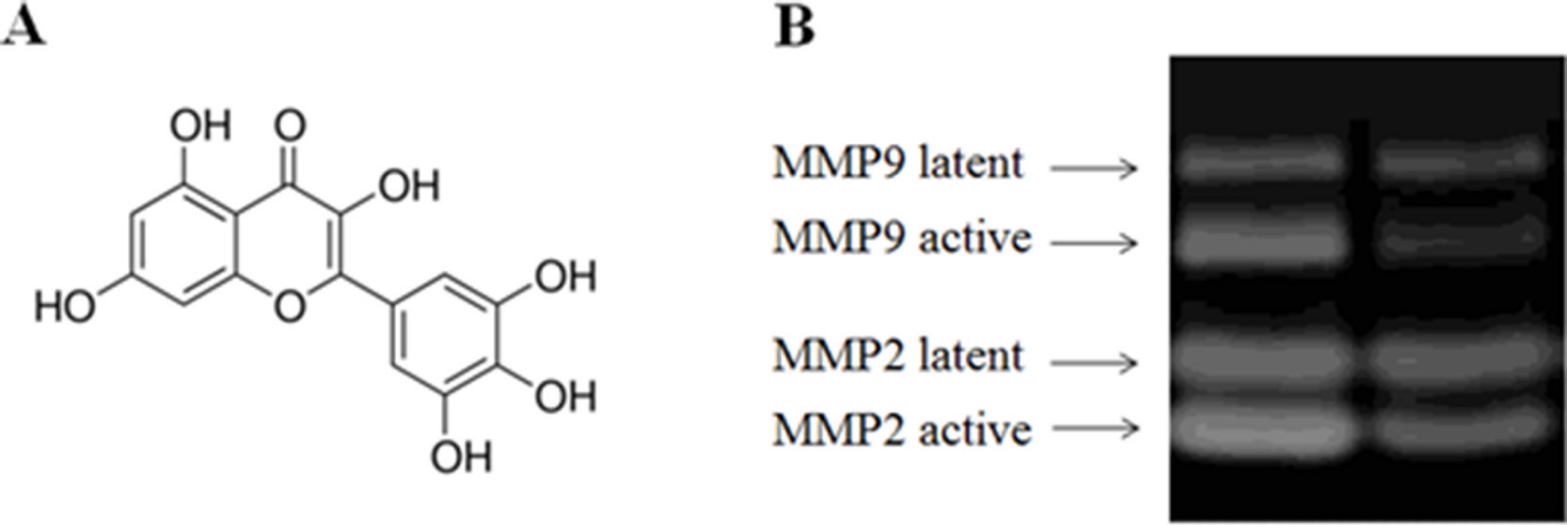
Increased expression level of active MMPs in fibroblasts treated by high glucose (30 mM) Legend: Lane 1 indicates the MMP zymography assay results from normal fibroblasts, and lane 2 shows the results from the fibroblasts treated with 30 mM glucose. These results indicated that the protein abundance levels of MMPs increased in glucose (30 mM) treated fibroblasts. The active forms of MMP2 and MMP9 increased significantly. Condition: The normal fibroblasts were cultured in DMEM cell culture containing 30 mM glucose. The cells were routinely cultured in a humidified atmosphere at 37°C, 5% (v/v) CO_2_, 95% (v/v) air, and they were used for experiments between passages 3 and 5 (80% confluence). The cultured media were assayed for MMP-1, MMP-2 and MMP-9 by casein and gelatin zymography. Zymographic images were digitized and quantified by scanning densitometry. Quantitative values for MMP-1, MMP-2 and MMP-9 were obtained and normalized against MMPs normalization standards.

**Figure 2:**
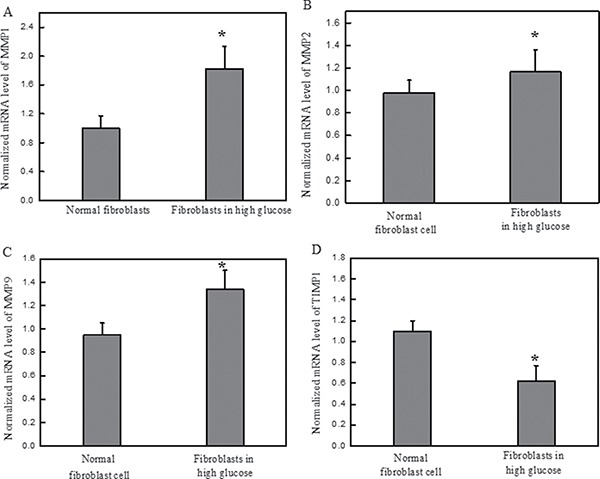
High glucose increased the mRNA expression levels of MMPs and decreased the expression level of TIMP1 on normal dermal fibroblasts Legend: (**A**) The normalized mRNA expression level of MMP1 in the normal fibroblasts cultured in high glucose media. (**B**) The normalized mRNA expression level of MMP2 in the normal fibroblasts cultured in high glucose media. (**C**) The normalized mRNA expression level of MMP9 in the normal fibroblasts cultured in high glucose media. (**D**) The normalized mRNA expression level of TIMP1 in the normal fibroblasts cultured in high glucose media. These results suggested that high glucose increases MMP levels, *P* < 0.05. The presence of high glucose alters the ratios between MMPs and TIMP1 which is a standard diagnostic of diabetic foot ulcerations. Condition: The normal dermal fibroblasts were cultured in DMEM media containing high glucose (30 mM) for four days. The mRNA expression levels of MMPs and TIMP1 were investigated by qRT-PCR, and the 2^−ΔΔCT^ method was used to analyze the relative changes in gene expression.

Dermal fibroblasts from healthy female were cultured in medium containing high glucose (30 mM) as mentioned. The zymography assay was carrier on in order to ascertain the changes of MMPs expression level upon the high glucose treatment. Result indicated the increase of active MMPs in high glucose-induced diabetic fibroblasts compared with healthy fibroblast cell (Figure 1). In addition, the mRNA expression levels of several biomarkers, including MMP1, MMP2, MMP9, TIMP1, procollagen type I and procollagen type III, were analyzed using qRT-PCR and then were compared with those from paralleled normal fibroblast cells. It showed that the mRNA levels of MMP1, MMP2 and MMP9 from normal fibroblasts in high glucose media were up-regulated significantly (Figure 2). The increased expression of MMPs in response to high glucose may reflect a reduction in the TIMP1 level. The results suggested that fibroblasts cultured in high glucose medium exhibit similar biological characteristics to diabetic fibroblasts.

### The effects of MDI 301 on the regulation of MMPs and TIMP1 in high glucose-induced diabetic fibroblasts

**Figure 3:**
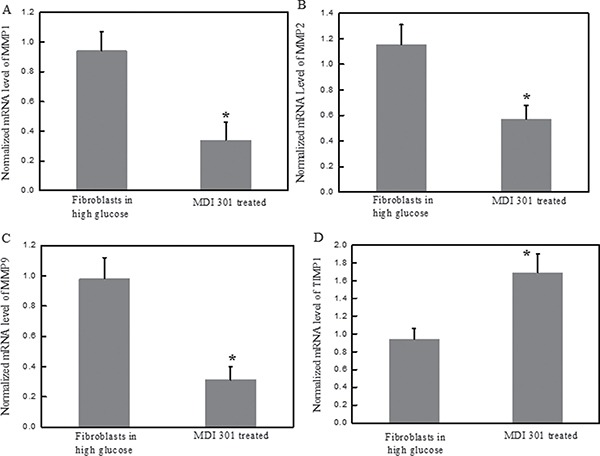
Effects of MDI 301 on the mRNA expression levels of MMPs in high glucose treated-fibroblast cells Legends: (**A**) The normalized mRNA expression level of MMP1 in high glucose treated-fibroblasts at the presence of 3 μM MDI 301. (**B**) The normalized mRNA expression level of MMP2 in high glucose treated-fibroblasts at the presence of 3 μM MDI 301. (**C**) The normalized mRNA expression level of MMP9 in high glucose treated-fibroblasts at the presence of 3 μM MDI 301. (**D**) The normalized mRNA expression level of TIMP1 in high glucose treated-fibroblasts at the presence of 3 μM MDI 301. These results showed a decrease in the mRNA expression levels of MMP1, MMP2 and MMP9 in the fibroblast cells treated with 3 μM MDI 301. After MDI 301 treatment, the mRNA expression level of MMP1 decreased by 67% (*P* < 0.05), MMP2 decreased by 51% (*P* < 0.05) and MMP9 decreased by 68% (*P* < 0.05). The mRNA expression level of TIMP1 was analyzed by qRT-PCR, and the data showed that the cells treated with MDI 301 had an increase of 72% (*P* < 0.05) compared to cells without MDI 301 incubation. Condition: The mRNA analysis kits for MMP1, MMP2 and MMP9 were supplied by Shanghai Sangon Institute of Biology, and the 2^−ΔΔCT^ method was used to analyze the relative changes in gene expression.

**Figure 4:**
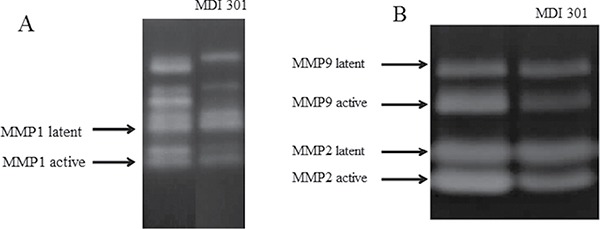
Effect of MDI 301 on the formation of active MMPs by MMP zymography assays Legend: (**A**) MMP zymography assay for MMP1. (**B**) MMP zymography assay for MMP2 and MMP9. These results indicated that MDI 301 is capable of inhibiting the formation of MMPs in fibroblasts treated by 30 mM glucose. Condition: The normal fibroblasts were cultured in DMEM cell culture containing high glucose (30 mM). The cells were routinely cultured in a humidified atmosphere at 37°C, 5% (v/v) CO_2_, and 95% (v/v) air and used for experiments between passages 3 and 5 (80% confluence). The cultured media were assayed for MMP-1, MMP-2 and MMP-9 by casein and gelatin zymography.

It was reported previously that MMP1, MMP2 and MMP9 are significantly over-expressed in diabetic fibroblasts and are important determinants of diabetic foot ulceration morbidity and exacerbation [3]. In this study, the effects of MDI 301 on high glucose-induced diabetic dermal fibroblasts were investigated, including the effects of MDI 301 on the expression levels of MMPs and TIMP1 in high glucose-induced diabetic fibroblasts after four days incubation with 3 μM MDI 301. qRT-PCR was used to analyze the mRNA expression levels of MMPs after incubation with MDI 301. As shown in Figure 3, the mRNA levels of MMP1, MMP2 and MMP9 were down-regulated by MDI 301 by 67%, 51% and 68% (*P* < 0.05), respectively. In addition, MDI 301 induced a 72% increase of TIMP1 mRNA expression as compared to high glucose-treated fibroblasts, as shown in Figure 3D (*P* < 0.05). The effects of MDI 301 on the active forms of MMPs were investigated by the MMP zymography assay, and the results showed a significant decrease in the levels of activated MMP1, MMP2 and MMP9, as shown in Figure 4A and 4B.

### Effects of MDI 301 on procollagen I and III of high glucose-induced diabetic fibroblasts

**Figure 5:**
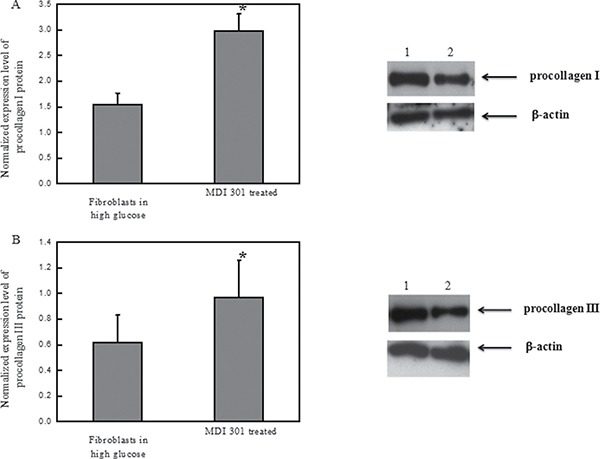
Effects of MDI 301 on procollagen I and III Legend: (**A** **left**) Normalized protein abundance of procollagen I in high glucose-treated fibroblasts treated with MDI 301; (**A right**). Western blotting analysis of procollagen I in high glucose-treated fibroblasts treated with MDI 301. Lane 1 shows the level of procollagen I in the fibroblasts treated with MDI 301. Lane 2 shows the level of procollagen I in the fibroblasts not treated with MDI 301. β-actin was employed as a control in this experiment. (**B** **left**) The normalized protein abundance of procollagen III in high glucose-treated fibroblasts treated with MDI 301. (**B right)** Western blotting analysis of procollagen III in high glucose-treated fibroblasts treated with MDI 301. Lane 1 shows the level of procollagen III in the fibroblasts treated with MDI 301. Lane 2 shows the level of procollagen III in the fibroblasts not treated with MDI 301. β-actin was employed as a control in this experiment. The western blotting results of procollagen I and III suggest that MDI 301 induces higher expression levels of procollagen I and III in high glucose-treated fibroblasts upon the treatment of MDI 301. The images were digitized and quantified by scanning densitometry. Quantitative values for procollagen I and procollagen III were obtained and being normalized against the normalization standards. The data shows that MDI 301 induced an increase of procollagen I (103%) and of procollagen III (53%) in the high glucose-treated fibroblasts treated with MDI 301, *P* < 0.05. Condition: The high glucose-treated fibroblasts were incubated with 3 μM MDI 301 for four days in a humidified atmosphere at 37°C, 5 % (v/v) CO_2_, and 95% (v/v) air and were used for experiments between passages 3 and 5 (80% confluence). The cultured media were assayed for procollagen I and procollagen III by western blotting assays.

Next, we examined the effects of MDI 301 on procollagen I and III by ELISA. Down-regulation of procollagen I and III is characteristic in diabetic foot ulcerations [12]. The protein abundance levels of procollagen I and III in high glucose-induced diabetic fibroblasts were increased significantly after 4 days incubation with 3 μM MDI 301. The levels of procollagen I and III showed a 103% and 53% increase, respectively, as shown in Figure 5.

To study whether MDI 301 could decrease the levels of catalase and superoxide dismutase, the two main enzymes involved in cellular oxidative stress, the mRNA expression levels of these two enzymes were investigated in the presence of MDI 301.

### Effects of MDI 301 on catalase and superoxide dismutase in high glucose-induced diabetic fibroblasts

**Figure 6:**
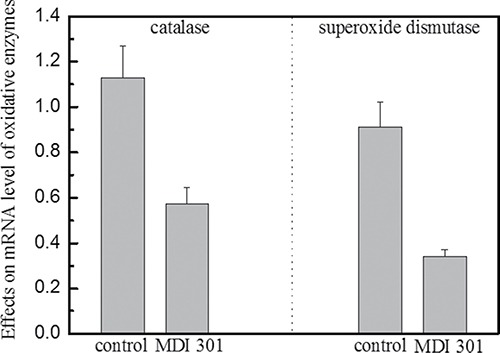
Effects of MDI 301 on catalase and superoxide dismutase involved in oxidative stress Legend: MDI 301 induced a 50% decrease in the mRNA expression level of catalase in high glucose-treated fibroblasts. In addition, MDI 301 induced a 64% decrease in the mRNA expression level of superoxide dismutase in high glucose-treated fibroblasts. These data indicate that the mRNA levels of both catalase and superoxide dismutase decrease in the presence of 3 μM MDI 301. Catalase and superoxide dismutase are involved in the induction of cellular oxidative stress which is attributed to the rise of MMP expression levels and activations. Condition: The cells were incubated with 3 μM MDI 301 for four days, and then the expression levels of catalase and superoxide dismutase were examined by qRT-PCR. The 2^−ΔΔCT^ method was used to analyze the relative changes in gene expression.

The high glucose-induced diabetic fibroblasts were incubated with 3 μM MDI 301 for four days, and then the mRNA expression levels of catalase and superoxide dismutase were analyzed. The results showed a significant decrease of catalase and superoxide dismutase levels. The expression of catalase and superoxide dismutase mRNAs decreased by 50% and 64%, respectively (Figure 6, *P* < 0.05). These results suggested that MDI 301 might act via balancing the oxidative stress level in diabetic fibroblasts.

## DISCUSSION

In diabetes, the lifetime risk of developing a foot ulcer is estimated to be 5%–15%. Approaches aimed at reducing the risk of ulceration or improving healing rates once ulceration has occurred are urgently required. The cause of ulceration in diabetic patients is multifactorial but may be accelerated by changes in the structure and function of the skin secondary to impaired fibroblast proliferation, decreased collagen synthesis and increased matrix metalloproteinases (MMPs) expression [13]. The expression of MMPs, including MMP1, MMP2 and MMP9, is significantly up-regulated in diabetic patients. Because oxidative stress presumably contributes to the up-regulation of MMPs [8], catalase and superoxide dismutase were considered to be involved in oxidative stress induction. MDI 301, a picolinic-acid-substituted 9-*cis* RA ester, was reported to exhibit beneficial effects on skin collagen synthesis and MMP activation in diabetic hairless rats [10].

In the current studies, we investigated the effect of high glucose on the formation of oxidative stress in normal fibroblasts obtained from healthy females and the biological effects of MDI 301 on these fibroblasts. Initially, normal fibroblasts obtained from healthy females were cultured in medium containing high glucose. These oxidants have been reported to be involved in the activation of latent MMPs, which results in many alterations in the developmental pathways in which MMPs are involved [8]. It has also been reported that high glucose levels might induce oxidative stress and apoptosis in neurons and diabetic patients [14–16]. We predicted that the oxidative stress induced by the presence of a high glucose concentration might be contributing to the oxidative unbalance and morbidity in diabetic patients. In our finding, it was found that the high glucose levels induced the up-regulation of MMPs (Figures 1 and 2). Results indicated that high glucose resulted in the increased expression of catalase and superoxide dismutase, which are involved in induction of cellular oxidative stress.

The MMP zymography assay was performed afterward in order to clarify the effects of MDI 301 on high glucose-induced diabetic fibroblasts. Results demonstrated that MDI 301 is capable of decreasing the mRNA expression levels of MMPs and up-regulating TIMP1 level on the high glucose treated fibroblasts (Figure 3). The results show that in the presence of 3 μM MDI 301, the expression levels of MMPs in high glucose-induced diabetic individuals were decreased to levels similar to those of cells derived from healthy females. Additionally, the MMP zymography assays showed that MDI 301 is capable of inhibiting the formation of the active forms of MMPs (Figure 4). The decreased levels of MMPs were correlated with the up-regulation of TIMP1. Combined with up-regulation of procollagen I and III (Figure 5), these results clearly suggested that MDI 301 is potent to alleviate the skin damage induced by high glucose. From Figure 6, it indicated that MDI 301 might regulate the MMPs and TIMP1 level depending on its activity on balancing the oxidative stress on the dermal fibroblasts treated by high glucose (30 mM).

## MATERIALS AND METHODS

### Materials

Healthy human dermal fibroblasts kindly supplied by Tianjin Medical University General Hospital were cultured in Dulbecco minimal Eagle's medium (DMEM) which contained a high glucose concentration (30 mM). The cells were routinely cultured in a humidified atmosphere at 37°C, 5% (v/v) CO_2_, and 95% (v/v) air, and cells between passages 3 and 5 (80% confluence) were used for the experiments. MDI 301 was purchased from Sigma Chemical Company (St. Louis, MO, USA).

### Quantitative real time PCR (qRT-PCR)

The mRNA analytical kits for detecting MMP1, MMP2, MMP9, TIMP1, catalase and superoxide dismutase were purchased from Shanghai Sangon Institute of Biology (Shanghai, China), and the 2^−ΔΔCT^ method was used to analyze the relative changes in gene expression.

### MMP assays

Medium from organ-cultured cells was assayed for abundance levels of MMP-1, MMP-2 and MMP-9 by casein and gelatin zymography, as described previously [17]. Zymographic images were digitized and quantified by scanning densitometry. Quantitative values for the expression level of MMP-1, MMP-2 and MMP-9 proteins were obtained and normalized against MMP normalization standards.

### ELISA assay for tissue inhibitor of metalloproteinase-1 (TIMP-1)

Culture media was assayed for abundance level of TIMP-1 by enzyme-linked immunosorbent assay (ELISA) using a commercially available assay kit (R&D Systems, Minneapolis, USA).

### Western blotting

Procollagen type I and III protein expression levels were detected by Western blotting. Antibodies were purchased from Santa Cruz Biotechnology Inc (California, US) and used according to the manufacturer's protocols.

### Statistical analysis

Student's *t-test* was employed for analyzing data. Unless otherwise stated, the results were reported as the mean ± standard error. *P* values less than 0.05 were considered significant.

## CONCLUSIONS

In conclusion, diabetic foot ulcerations exhibit functional defects which may predispose individuals to fragility and ulceration. We presumed that high glucose might be one of the important factors for the formation of diabetic skin damage. It was confirmed that the induction of oxidative stress, which could be influenced by MDI 301, is associated with high glucose levels. It was indicated that MDI 301 might to ameliorating structural skin defects in these high-risk patients.
